# *Mycobacterium* genus and tRNA arrays

**DOI:** 10.1590/0074-02760180443

**Published:** 2019-05-13

**Authors:** Sergio Mascarenhas Morgado, Ana Carolina Paulo Vicente

**Affiliations:** 1 Fundação Oswaldo Cruz-Fiocruz, Instituto Oswaldo Cruz, Laboratório de Genética Molecular de Microrganismos, Rio de Janeiro, RJ, Brasil

**Keywords:** tRNA array, Mycobacterium, Mycolicibacterium, Mycolicibacillus, Mycobacteroides, Actinobacteria

## Abstract

The presence of tRNA array, a region with high tRNA gene number and density, has been demonstrated in *Mycobacterium* genus. However, a recent phylogenomic study revealed the existence of five distinct monophyletic groups (genera) within this genus. Considering this new scenario, and based on *in-silico* analyses, we have identified and characterised the abundance and diversity of tRNA array units within *Mycobacterium*, *Mycolicibacterium* gen. nov., *Mycolicibacillus* gen. nov., and *Mycobacteroides* gen. nov. The occurrence and prevalence of tRNA arrays among the genera belonging to *Actinobacteria* indicate their possible role in the organismal fitness.

The tRNA genes are generally distributed along the genomes and are eventually arranged in small clusters (up to five genes), as seen in mitogenomes.^1^ ^,^ ^2^ In contrast to these small clusters, genomic regions with a high number (from 19 to 39 tRNA genes) and density of tRNA genes have been identified and characterised in archaea, eukaryotes, bacteria, and viruses.^3^ ^,^ ^4^ ^,^ ^5^ ^,^ ^6^ ^,^ ^7^ ^,^ ^8^ ^,^ ^9^ ^,^ ^10^ As far as the bacteria are concerned, it was initially thought that such structures would occur only in a few phyla, particularly *Firmicutes*.^8^ However, tRNA arrays have been revealed to be abundant among *Mycobacterium*; they have been found in chromosomes and/or plasmids of several species, including fast- and slow-growing species.^9^ Recently, a study proposed a reclassification for the *Mycobacterium* genus into five clades; one of them has been associated with the older *Mycobacterium* genus and four new clades have been suggested: *Mycolicibacterium* gen. nov., *Mycolicibacter* gen. nov., *Mycolicibacillus* gen. nov., and *Mycobacteroides* gen. nov.^11^ In the face of this reclassification and due to the importance of this phylum, which encompasses organisms of agricultural, biotechnological, clinical, and ecological importance, we questioned if tRNA arrays occurred in all five clades (genera), particularly in *Mycobacterium*. In order to verify the presence of tRNA arrays in these five proposed clades (genera), we screened the representative genomes belonging to them. The tRNA arrays were identified and characterised based on a previous methodology for *Mycobacterium* genus.^9^ A phylogenetic tree, based on the core genome of these bacteria, was build (cgMLSA) considering the representative genomes harbouring tRNA arrays, from each of the proposed clade (36 genomes in total). The cgMLSA analysis was performed by GET_HOMOLOGUES v3.05^12^ using parameters of minimum coverage of ≥ 70% and identity of ≥ 40%. The sequences were aligned using MAFFT v7.271^13^ and trimmed using trimAL v1.2.^14^ A neighbour joining tree, based on 806 concatenated genes (~831 kb), was constructed using Seaview v4.7^15^ with 1000 bootstrap replicates and edited using iTOL.^16^ This analysis revealed that the tRNA arrays occur in species from *Mycobacterium* genus as well as in all new proposed genera, except *Mycolicibacter* gen. nov. (Figure). In fact, there is a bias concerning this new genus due to low number of genomes/drafts available in NCBI; therefore, the occurrence of the tRNA arrays cannot be completely ruled out. The arrays presented a tRNA repertoire that varied from 16 to 20 isotypes, most of them presenting the 20 universal tRNA isotypes. Despite the large tRNA repertoire provided by the arrays, these tRNA isotypes are redundant compared to those of non-arrayed tRNAs. However, few genomes (genome labels highlighted in bold in Figure) had an increment in the number of isoacceptor species (one or two), being the isoacceptor tRNA-Thr^AGU^ the one present in all these genomes, except in *M. koreense* KCTC 19819, that presented tRNA-His^AUG^ isoacceptor. Most of the arrayed tRNAs seem to be functional as only a few genes (one or two per array in 11/21 genomes harbouring tRNA array) were annotated as pseudogenes (isotype letters highlighted in bold in Figure). Interestingly, similar tRNA isotype organisation of some tRNA arrays was identified in genomes from *Mycobacterium* species and from the newly proposed genera. In a previous study, based on the tRNA gene isotype organisation, the tRNA arrays from *Mycobacterium* genomes were characterised in three groups and a singleton,^9^ as also has been observed in this study. The tRNA array group 2 (represented by squares in the Figure) is present in the genomes from *Mycolicibacterium* gen. nov. and *Mycobacteroides* gen. nov., while the tRNA array group 1 (represented by circles in the Figure) and the tRNA array singleton (represented by triangles in the Figure) are exclusively observed in the genomes from *Mycobacteroides* gen. nov. and *Mycolicibacillus* gen. nov., respectively. Taken together, these results show the presence of tRNA arrays in the *Mycobacterium* genus even after the its reclassification as well as in other *Actinobacteria* genera; this is in contrast with the previous study that revealed lower prevalence of such structures in this phylum.^8^ Moreover, the dispersion of tRNA arrays with the same isotype organisation among different species and genera corroborates the hypothesis of their association with mobile elements. Indeed, tRNA arrays were associated with horizontal gene transfer events mediated by the plasmids and bacteriophages.^8^ ^,^ ^9^ Particularly, tRNA gene clusters have been shown to be prevalent among mycobacteriophages.^5^ ^,^ ^17^ Even though the role of these elements is still debated,^3^ ^,^ ^6^ ^,^ ^18^ their occurrence and prevalence among eukaryotes, prokaryotes, and viruses indicate a possible positive implication in the organismal fitness.

**Figure:**
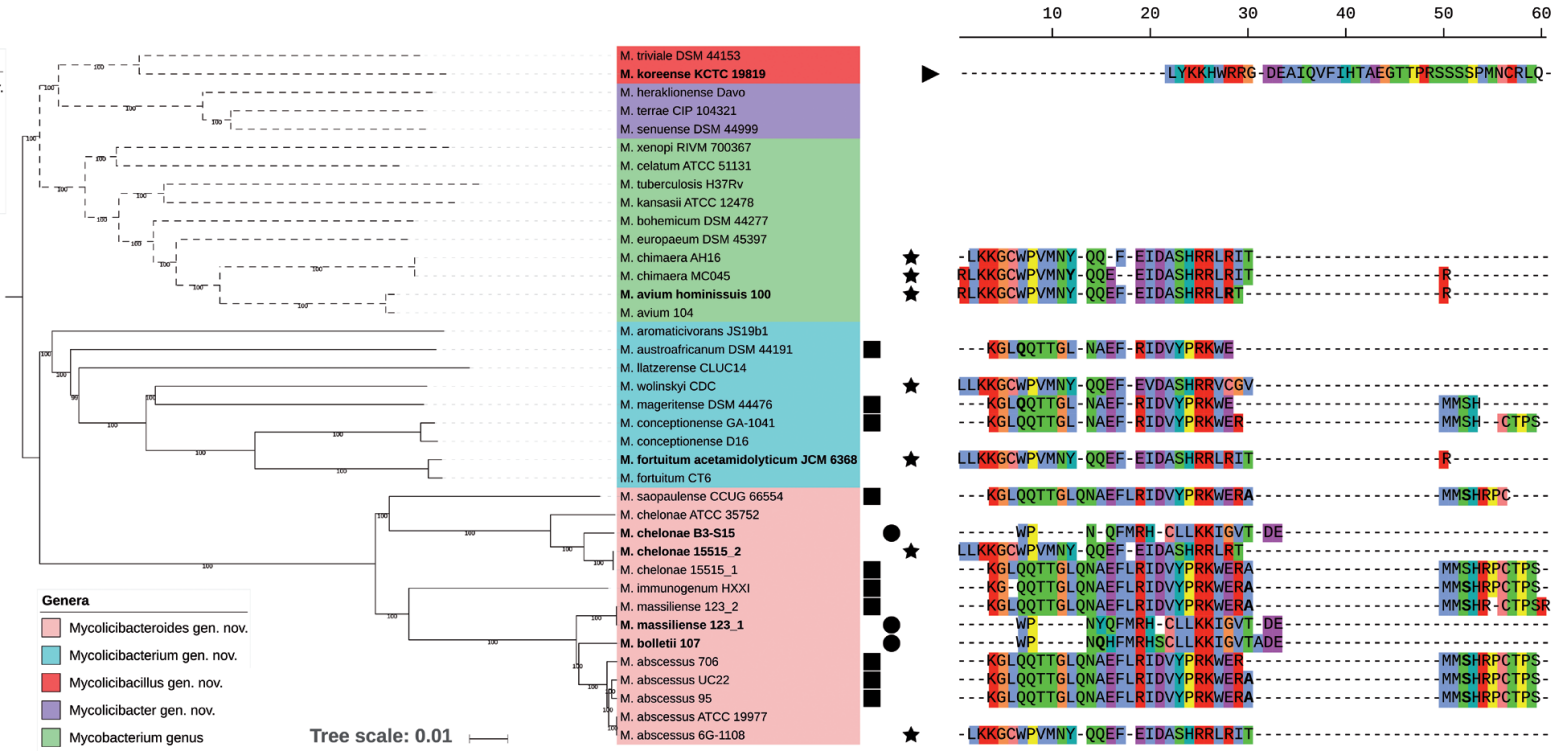
Phylogenetic tree of *Mycobacterium*, *Mycolicibacterium* gen. nov., *Mycolicibacter* gen. nov., *Mycolicibacillus* gen. nov., and *Mycobacteroides* gen. nov. (left side). The genera are discriminated by the background colour and the dotted branches correspond to the slow-growing species. The genomes highlighted in bold represent those with an increase in the isoacceptor species. The symbols indicate tRNA array group: circle, group 1; square, group 2; star, group 3; triangle, singleton. The tRNA isotype organisation of each array is represented using the single-letter amino acid code (right side). The isotypes in bold correspond to the genes annotated as pseudogenes.
